# Do certain jobs increase a woman’s risk of pregnancy loss?

**Published:** 2023-02

**JANUARY 21, 2023** - In an analysis of 2010–2019 information on more than 1.8 million employed and non-employed pregnant women in South Korea, certain occupations were linked with higher risks of miscarriage and stillbirth.

For the study, which is published in the Journal of Occupational Health, investigators calculated risks for three adverse outcomes: early abortive outcomes (miscarriage, ectopic pregnancy, and molar pregnancy), stillbirth, and no live birth (pregnancy with no record of live birth thereafter, which include early abortive outcomes and stillbirth).

Overall, 18.0%, 0.7%, and 39.8% of pregnancies ended in early abortive outcomes, still-births, and no live births, respectively. The risk of early abortive outcomes and stillbirths was higher in non-employed women than in employed women, while no live births were more frequent in employed women.

Women in the health and social work industry had the highest risk of no live births. Higher risks of no live births were also observed in the manufacturing, wholesale/retail trade, education, and public/social/personal service occupations. Manufacturing jobs and health/social work were associated with higher risks of early abortive outcomes compared with financial and insurance jobs.

“The good news is that the Ministry of Employment and Labor of South Korea is now revising the Industrial Accident Compensation Insurance Act to cover all the abortive outcomes in pregnant women workers. Our study contributed to the amendment of this Act, as we presented the impact of the occupational environment on adverse pregnancy outcomes,” said corresponding author Jung-won Yoon, MD, of the National Medical Center in Seoul.

*URL upon publication: https://onlinelibrary.wiley.com/doi/10.1002/1348-9585.12380*

*Full citation: “Risk of adverse pregnancy outcomes by maternal occupational status: A national population-based study in South Korea.” Chae-Bong Kim, Seung-Ah Choe, Taemi Kim, Myoung-Hee Kim, Jia Ryu, Jeong-Won Oh, Jung-won Yoon. J Occup Health; Published Online: 25 January 2023 (DOI: 10.1002/1348-9585.12380).*

*Copyright © 2019 The Cochrane Collaboration. Published by John Wiley & Sons, Ltd., reproduced with permission.*

